# Relationship between baseline D-dimer and prognosis in Japanese patients with venous thromboembolism: Insights from the J’xactly study

**DOI:** 10.3389/fcvm.2023.1074661

**Published:** 2023-02-09

**Authors:** Shohei Migita, Yasuo Okumura, Ikuo Fukuda, Mashio Nakamura, Norikazu Yamada, Morimasa Takayama, Hideaki Maeda, Takeshi Yamashita, Takanori Ikeda, Makoto Mo, Tsutomu Yamazaki, Atsushi Hirayama

**Affiliations:** ^1^Division of Cardiology, Department of Medicine, Nihon University School of Medicine, Tokyo, Japan; ^2^Department of Cardiology, Keimeikai Yokawa Hospital, Miki, Japan; ^3^Nakamura Medical Clinic, Kuwana, Japan; ^4^Department of Cardiology, Kuwana City Medical Center, Kuwana, Japan; ^5^Department of Cardiology, Sakakibara Heart Institute, Fuchu, Japan; ^6^Department of Heart and Vascular Center, Ukima Central Hospital, Tokyo, Japan; ^7^Department of Cardiovascular Medicine, The Cardiovascular Institute, Tokyo, Japan; ^8^Department of Cardiovascular Medicine, Toho University Faculty of Medicine, Tokyo, Japan; ^9^Department of Cardiovascular Surgery, Yokohama Minami Kyosai Hospital, Yokohama, Japan; ^10^Innovation and Research Support Center, International University of Health and Welfare, Tokyo, Japan; ^11^Department of Cardiology, Osaka Police Hospital, Osaka, Japan

**Keywords:** anticoagulant, bleeding, recurrence, rivaroxaban, venous thromboembolism

## Abstract

**Background:**

D-dimer is a biomarker of fibrin production and degradation, and changes in D-dimer concentration suggest fibrin clot formation, which is associated with thromboembolism and hypercoagulable states. Thus, an elevated D-dimer concentration could be a useful prognostic predictor for patients with venous thromboembolism (VTE).

**Methods and results:**

In this subanalysis of the J’xactly study, a prospective multicenter study conducted in Japan, we examined the clinical outcomes of 949 patients with VTE stratified by baseline D-dimer concentration. The median D-dimer concentration was 7.6 μg/ml (low D-dimer group: <7.6 μg/ml [*n* = 473, 49.8%]; high D-dimer group: ≥7.6 μg/ml [*n* = 476, 50.2%]). The mean age of the patients was 68 years, and 386 patients (40.7%) were male. Compared with the low D-dimer group, the high D-dimer group had more frequent pulmonary embolism with or without deep vein thrombosis (DVT), proximal DVT, atrial fibrillation, or diabetes mellitus, and underwent intensive treatment with 30 mg/day rivaroxaban. The incidence of composite clinically relevant events (recurrence or exacerbation of symptomatic VTE, acute coronary syndrome [ACS], ischemic stroke, death from any cause, or major bleeding) was higher in the high D-dimer group than in the low D-dimer group (11.1% vs. 7.5% per patient-year; hazard ratio, 1.46; 95% confidence interval, 1.05–2.04; *p* = 0.025). There was no significant difference between the high and low D-dimer groups in the incidence of VTE (2.8% vs. 2.5% per patient-year, respectively; *p* = 0.788), ACS (0.4% per patient-year vs. not observed, respectively; *p* = 0.078), or major bleeding (4.0% vs. 2.1% per patient-year, respectively; *p* = 0.087), but there was a significant difference in the incidence of ischemic stroke (1.0% per patient-year vs. not observed, respectively; *p* = 0.004).

**Conclusion:**

Elevated D-dimer concentration may be an important prognostic predictor in Japanese patients with VTE.

**Clinical Trial Registration**: UMIN CTR, UMIN000025072 (https://www.umin.ac.jp/ctr/index.htm).

## 1. Introduction

Venous thromboembolism (VTE) is a common, acute cardiovascular disorder that encompasses both deep vein thrombosis (DVT) and pulmonary embolism (PE) (1, 2), and it is a major medical concern worldwide (3). Death occurs in approximately 6% of cases of DVT and in approximately 12% of cases of PE within 1 month of diagnosis (4).

Blood D-dimer concentration is a biomarker of fibrin production and degradation. Changes in D-dimer concentration suggest that fibrinolysis is in progress, potentially indicating fibrin clot formation associated with thromboembolism and hypercoagulable states (5, 6). In fact, D-dimer concentration within the normal range is used to rule out the diagnosis of DVT and PE in patients with a low clinical probability of VTE (7, 8). Moreover, an increase in D-dimer concentration after discontinuation of anticoagulant therapy is an indicator of DVT recurrence and can be used as a reference to determine the duration and termination of anticoagulant therapy (9).

Changes in D-dimer concentration are seen in aging and hospitalized patients, as well as in pregnant women (10). A previous report showed that patients with high D-dimer concentrations exceeding 5 μg/ml are often complicated by sepsis and malignant tumor (11). However, it is unclear whether an elevated baseline D-dimer concentration can directly serve as a clinical index item to predict VTE prognosis.

In this study, we aimed to elucidate the association between the baseline D-dimer concentration at admission and the clinical outcomes of patients with VTE using data from the Japanese Registry of RivaroXAban Effectiveness and Safety for the Prevention of Recurrence in Patients with Deep Vein Thrombosis and PuLmonarY Embolism (J’xactly study) (12, 13), in which 1,039 patients with acute symptomatic/asymptomatic DVT or PE with or without DVT who underwent treatment with rivaroxaban were enrolled (12, 13).

## 2. Materials and methods

### 2.1. Study design and participants

The full details of the study design, data collection process, and baseline characteristics of the study population have been reported previously (12, 13). The J’xactly study was a multicenter, prospective, observational cohort study in which patients diagnosed with acute symptomatic/asymptomatic DVT, PE, or both, and who were prescribed rivaroxaban for the treatment and prevention of VTE, were enrolled from December 2016 to April 2018.

The key exclusion criteria were contraindications to rivaroxaban; the presence of chronic thromboembolic pulmonary hypertension (CTEPH), except for CTEPH plus acute PE or DVT; and active bleeding. All patients provided written informed consent for study participation.

The J’xactly study was conducted in accordance with the principles of the Declaration of Helsinki and with all applicable legal and regulatory requirements in Japan. The protocol and related documentation were reviewed and approved by the Institutional Review Board of Nihon University Itabashi Hospital. All participating institutions also provided ethics approval. In addition, an independent data and safety monitoring committee reviewed all of the study data. The study was registered in the University hospital Medical Information Network Clinical Trials Registry as UMIN000025072.

All eligible patients were enrolled in the study within 3 weeks of starting treatment with rivaroxaban, and data were collected until the end of the follow-up period (November 2019), regardless of whether rivaroxaban was continued, discontinued, or terminated according to the patient’s preference or the physician’s discretion. The 1,016 patients analyzed were stratified by the initial dose of rivaroxaban (standard dosage: 30 mg/day; underdosages: 20, 15, or 10 mg/day). In addition, patients were stratified according to the presence of DVT only or PE with or without DVT. DVT was classified by localization of the thrombus and classified as either proximal (thrombus located proximal to or involving the popliteal vein) or distal (thrombus located distal to the popliteal vein). PE severity was stratified according to Japanese guidelines (2) as either cardiac arrest or collapse, massive, sub-massive, or non-massive PE, other than those described above.

### 2.2. Data collection

Of the 1,016 patients evaluable by modified intention-to-treat (mITT), 949 patients with available D-dimer concentrations at baseline (at rivaroxaban initiation) were included. In addition, clinical outcomes were evaluated by dividing the patients into two groups based on the median baseline D-dimer concentration of 7.6 μg/ml. The efficacy analysis population included 949 patients, and the safety analysis population included 950 patients.

### 2.3. Outcomes

As previously described (12, 13), the primary effectiveness outcome was recurrence or aggravation of symptomatic VTE during the follow-up period. VTE was defined according to established diagnostic criteria (14, 15). The primary safety outcome was a major bleeding event that occurred during the treatment period and up to 2 days after rivaroxaban discontinuation. Major bleeding was defined according to the International Society on Thrombosis and Hemostasis criteria (16).

The secondary outcomes included recurrence or aggravation of symptomatic DVT and PE, death from any cause, death related to VTE and cardiovascular disease (CVD), vascular events (acute coronary syndrome [ACS] or ischemic stroke), and non-major bleeding. Clinically relevant events were also evaluated as a composite outcome, in which each component (recurrent VTE, ACS, ischemic stroke, death from any cause, and major bleeding events) was weighted equally. An independent, blinded clinical events committee adjudicated the outcomes.

### 2.4. Statistical analysis

The mITT population was used for effectiveness calculations; this included all enrolled patients except those who were excluded from study participation. The on-treatment population was used for safety assessments; this included all patients who were treated with at least one dose of rivaroxaban. Continuous variables are reported as the mean ± standard deviation, and categorical variables are reported as the number and percentage of patients. The high and low D-dimer groups were compared using the *t*-test for continuous variables and the Chi-square test for categorical variables. The Kaplan–Meier method was used to estimate the cumulative event rates, with incidence rates in each treatment group shown as percentages per patient-year. The Cox proportional hazards regression model was used to compare the outcomes between the two groups, with the results expressed as hazard ratios (HRs) with 95% confidence intervals (CIs). A landmark analysis was based on extended treatment with rivaroxaban. Surviving patients who were free from the primary effectiveness and safety outcomes at the landmark cut-off (30 days after the initial treatment) were included in the landmark analysis for the primary effectiveness outcome, death from any cause, safety outcomes, and clinically relevant events. All statistical analyses were performed using SAS software, version 9.4 for Windows (SAS Institute, Inc., Cary, NC, United States). A *p* < 0.05 was considered statistically significant.

## 3. Results

### 3.1. Patients

**Figure 1:**
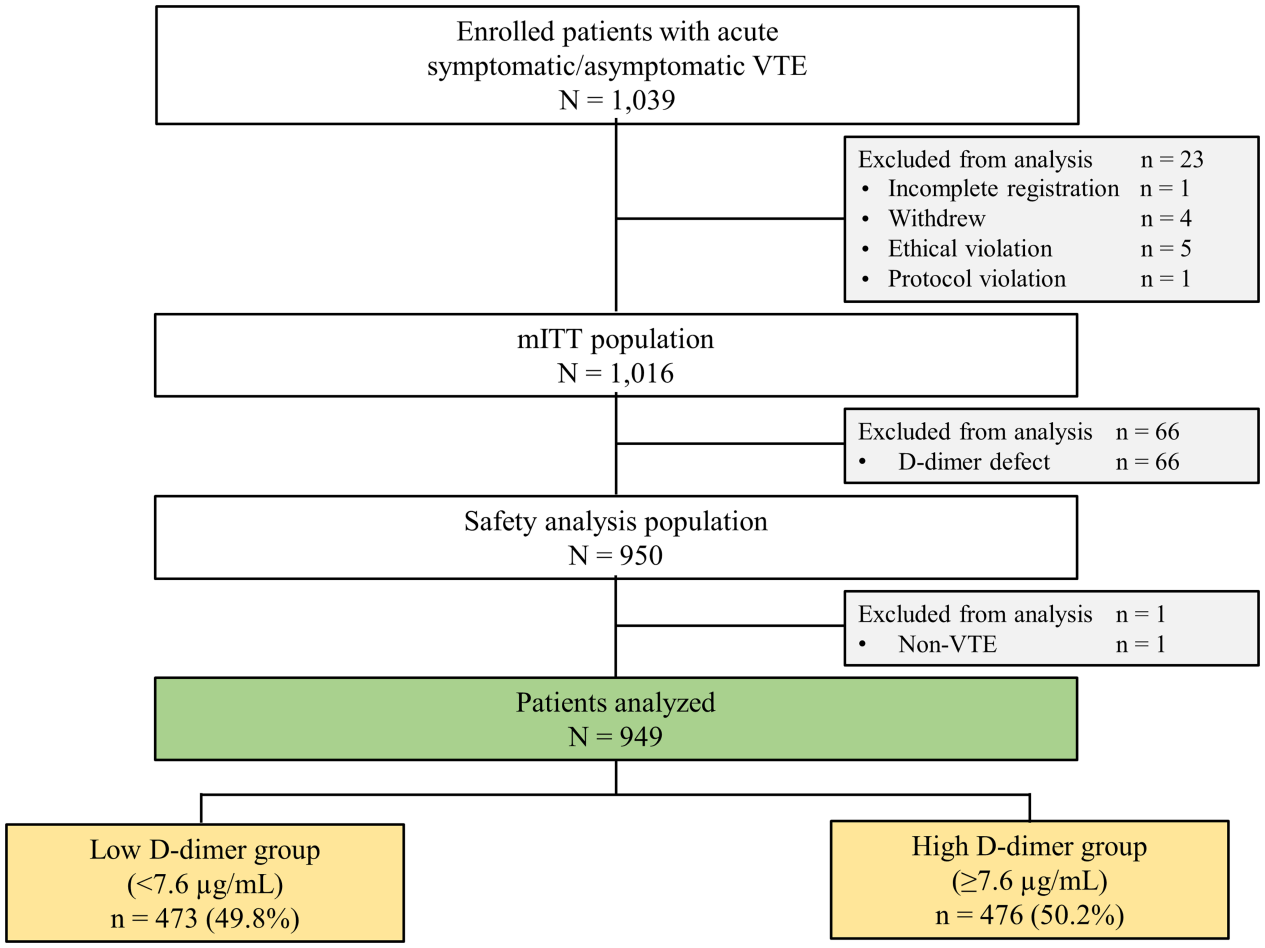
Flowchart of patient selection and stratification by D-dimer concentration. mITT, modified intention-to-treat; VTE, venous thromboembolism.

Among the 1,016 mITT patients, 949 patients with available baseline D-dimer concentrations were included. The median D-dimer concentration was 7.6 μg/ml. Based on this median value, patients were divided into the low D-dimer group (<7.6 μg/ml [*n* = 473, 49.8%]) and the high D-dimer group (≥7.6 μg/ml [*n* = 476, 50.2%]) (Figure 1). The on-treatment population included the same 950 patients as the mITT population. Patients were followed up until November 2019. The median follow-up period was 21.2 months (interquartile range, 18.1–24.0 months), and the percentage of patients lost to follow-up was 0.9% (*n* = 9).

### 3.2. Patient characteristics according to baseline D-dimer concentration: DVT and PE with/without DVT

The baseline characteristics of patients according to baseline D-dimer concentration are summarized in Table 1, and the distribution of baseline D-dimer concentration is shown in Supplementary Figure S1. Patients in the low D-dimer group were significantly younger than those in the high D-dimer group (66.7 ± 15.0 vs. 69.2 ± 14.4 years, respectively; *p* = 0.007). The low D-dimer group had a lower proportion of patients with proximal DVT (45.5% vs. 64.5%; *p* < 0.001), PE (31.9% vs. 51.5%; *p* < 0.001), atrial fibrillation (1.5% vs. 4.0%; *p* = 0.027), and diabetes mellitus (9.1% vs. 14.1%; *p* = 0.019). Comorbidities in patients with VTE, such as active cancer (18.2% vs. 19.1%, respectively; *p* = 0.739), previous VTE (8.7% vs. 7.6%, respectively; *p* = 0.55), and previous stroke (5.9% vs. 7.8%, respectively; *p* = 0.304), were not significantly different between the low and high D-dimer groups.

**Table 1:** Baseline characteristics of patients stratified by baseline D-dimer concentration.

Variable	Total *n* = 949	Baseline D-dimer concentration		*p* value
		Low *n* = 473	High *n* = 476	
**Age, years**	68.0 ± 14.7	66.7 ± 15.0	69.2 ± 14.4	0.007
≥75 years	362 (38.1)	163 (34.5)	199 (41.8)	0.033
Female sex	563 (59.3)	280 (59.2)	283 (59.5)	0.947
**Risk factor**				
Inactivity	342 (36.0)	140 (29.6)	202 (42.4)	<0.001
Injury	89 (9.4)	36 (7.6)	53 (11.1)	0.075
Surgery	241 (25.4)	107 (22.6)	134 (28.2)	0.053
Active cancer	177 (18.7)	86 (18.2)	91 (19.1)	0.739
Thrombophilia	36 (3.8)	16 (3.4)	20 (4.2)	0.611
Previous VTE	77 (8.1)	41 (8.7)	36 (7.6)	0.554
**CrCl, mL/min**	77.9 ± 36.3	78.9 ± 34.8	77.5 ± 38.1	0.185
<30	9 (0.9)	6 (1.3)	3 (0.6)	0.327
≥30 to <50	195 (20.5)	80 (16.9)	115 (24.2)	0.024
≥50 to <80	340 (35.8)	171 (36.2)	169 (35.5)	0.373
≥80	368 (38.8)	183 (38.7)	185 (38.9)	0.500
**Body weight, kg**	60.3 ± 14.1	60.2 ± 13.9	60.8 ± 14.4	0.675
<50 kg	204 (21.5)	95 (20.1)	109 (22.9)	0.563
Body mass index, kg/m^2^	23.8 ± 4.2	23.6 ± 4.1	24.1 ± 4.3	0.243
Heart rate, beats per minute	82.7 ± 17.2	79.8 ± 15.5	85.7 ± 18.2	<0.001
SpO_2_, %	96.1 ± 3.7	96.4 ± 3.9	95.9 ± 3.5	0.004
**Medical history**				
Previous stroke	65 (6.8)	28 (5.9)	37 (7.8)	0.304
Coronary artery disease	42 (4.4)	25 (5.3)	17 (3.6)	0.211
Hypertension	354 (37.3)	162 (34.2)	192 (40.3)	0.060
Diabetes mellitus	110 (11.6)	43 (9.1)	67 (14.1)	0.019
Heart failure	31 (3.3)	14 (3.0)	17 (3.6)	0.716
Atrial fibrillation	26 (2.7)	7 (1.5)	19 (4.0)	0.027
Chronic heart and lung disease	42 (4.4)	19 (4.0)	23 (4.8)	0.636
**Concomitant medications**				
Antiplatelet agents	97 (10.2)	54 (11.4)	43 (9.0)	0.240
NSAIDs	181 (19.1)	75 (15.9)	106 (22.3)	0.013
Estrogen preparations	23 (2.4)	13 (2.7)	10 (2.1)	0.535
Anticancer agents	78 (8.2)	33 (7.0)	45 (9.5)	0.194
Hospitalization	570 (60.1)	231 (48.8)	339 (71.2)	<0.001
**Duration of hospitalization**				
1–4 days	24 (2.5)	8 (1.7)	16 (3.4)	0.529
5–7 days	51 (5.4)	22 (4.7)	29 (6.1)	0.765
8–14 days	160 (16.9)	70 (14.8)	90 (18.9)	0.343
15–21 days	111 (11.7)	38 (8.0)	73 (15.3)	0.161
22–28 days	57 (6.0)	21 (4.4)	36 (7.6)	0.573
≥29 days	167 (17.6)	72 (15.2)	95 (20.0)	0.454
**DVT**	863 (90.9)	426 (90.1)	437 (91.8)	0.367
Proximal	505 (53.2)	215 (45.5)	307 (64.5)	<0.001
Distal	341 (35.9)	211 (44.6)	130 (27.3)	<0.001
Symptomatic DVT	568 (59.9)	283 (59.8)	285 (59.9)	0.720
**PE**	394 (41.5)	151 (31.9)	243 (51.5)	<0.001
Cardiac arrest or collapse	6 (0.6)	1 (0.2)	5 (1.1)	0.413
Massive	15 (1.6)	6 (1.3)	9 (1.9)	1
Sub-massive	121 (12.8)	43 (9.1)	78 (16.4)	0.501
Non-massive	230 (24.2)	90 (19.0)	140 (29.4)	0.753
Unknown	22 (2.3)	11 (2.3)	11 (2.3)	0.265
Symptomatic PE	209 (22.0)	75 (15.9)	134 (28.2)	0.301
**Prior treatment for VTE**	316 (33.3)	125 (26.4)	191 (40.1)	<0.001
Anticoagulation therapy	248 (26.1)	98 (20.7)	150 (31.5)	<0.001
Inferior vena cava filter	87 (9.2)	21 (4.4)	66 (13.9)	<0.001
Thrombolytic therapy	45 (4.7)	21 (4.4)	24 (5.0)	0.760
Catheterization	11 (1.2)	3 (0.6)	8 (1.7)	0.224
Pulmonary thrombus removal	1 (0.1)	0 (0.0)	1 (0.2)	1
PCPS	3 (0.3)	1 (0.2)	2 (0.4)	1
Other	23 (2.4)	16 (3.4)	7 (1.5)	0.060
**Initial rivaroxaban treatment**				
*Dose, mg/day*				<0.001
30	647 (68.2)	289 (61.1)	358 (75.2)	
20	20 (2.1)	5 (1.1)	15 (3.2)	
15	248 (26.1)	160 (33.8)	88 (18.5)	
10	34 (3.6)	19 (4.0)	15 (3.2)	
**Initial intensified therapy with 30-mg/day rivaroxaban**				
Treatment duration, days				
Mean ± SD	357.9 ± 268.7	344.5 ± 271.4	369.8 ± 263.4	0.145
Median (IQR)	282 (106–619)	241 (98–617)	332 (130–619)	0.125

Data are presented as *n* (%) or mean ± SD. CrCl, creatinine clearance; DVT, deep vein thrombosis; IQR, interquartile range; NSAIDs, non-steroidal anti-inflammatory drugs; PCPS, percutaneous cardiopulmonary support; PE, pulmonary embolism; SD, standard deviation; SpO_2_, oxygen saturation; VTE, venous thromboembolism.

The low D-dimer group had a lower proportion of hospitalized patients (48.8% vs. 71.2%; *p* < 0.001), a lower heart rate (79.8 ± 15.5 vs. 85.7 ± 18.2 beats per minute; *p* < 0.001), and a higher oxygen saturation (SpO_2_) (96.4% ± 3.9% vs. 95.9% ± 3.5%; *p* = 0.004), but there were no significant differences in the period of hospitalization, PE severity, and the proportion of symptomatic patients. A higher proportion of patients in the high D-dimer group underwent initial intensified therapy with rivaroxaban 30 mg/day (75.2% vs. 61.1%; *p* < 0.001), but there was no significant difference in the duration of treatment between the low and high D-dimer groups until the last dose (345 ± 271 vs. 370 ± 263 days, respectively; *p* = 0.13).

### 3.3. Clinical outcomes according to baseline D-dimer concentration: DVT and PE with/without DVT

**Figure 2:**
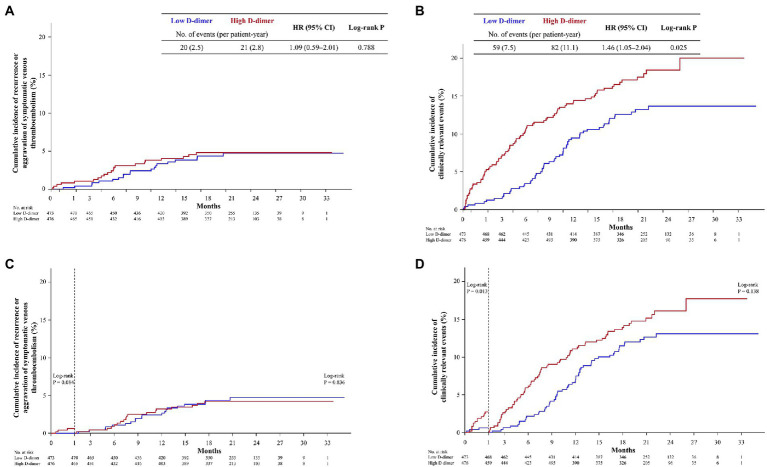
Kaplan–Meier curves showing the cumulative incidence of **(A)** recurrence or aggravation of symptomatic VTE and **(B)** clinically relevant events. The rate of recurrence or aggravation of symptomatic VTE tended to be higher within 30 days of starting rivaroxaban therapy in both the high (red) and low (blue) D-dimer groups **(C)**. For the composite of clinically relevant events, a significant difference was observed between the high D-dimer group (red) and the low D-dimer group (blue) within 30 days **(D)**. CI, confidence interval; HR, hazard ratio; VTE, venous thromboembolism.

The clinical outcomes of the high and low D-dimer groups are shown in Table 2. The high D-dimer group tended to have a higher incidence of major bleeding (4.0% vs. 2.1% per patient-year; *p* = 0.087) and ACS (0.4% per patient-year vs. not observed; *p* = 0.078) than the low D-dimer group, although there were no significant differences. The incidence of ischemic stroke was significantly higher in the high D-dimer group than in the low D-dimer group (1.0% per patient-year vs. not observed; *p* = 0.004). The Kaplan–Meier analyses indicated no significant differences between the high and low D-dimer groups in the cumulative incidences of recurrence or aggravation of symptomatic VTE (2.8% vs. 2.5% per patient-year, respectively; *p* = 0.788) (Figure 2A). As a result of the higher incidence of ischemic stroke in the high D-dimer group, the incidence of composite clinical events was significantly higher in the high D-dimer group than in the low D-dimer group (11.1% vs. 7.5% per patient-year; HR, 1.46; 95% CI, 1.05–2.04; *p* = 0.025) (Figure 2B).

**Table 2:** Clinical outcomes of patients stratified by baseline D-dimer concentration.

	Baseline D-dimer concentration				HR (95% CI)	*p* value
	Low *n* = 473		High *n* = 476			
	*n* (%)	Per patient-year	*n* (%)	Per patient-year		
Recurrence or aggravation of symptomatic VTE	20 (4.2)	2.5	21 (4.4)	2.8	1.09 (0.59–2.01)	0.788
Recurrence or aggravation of symptomatic PE	9 (1.9)	1.1	9 (1.9)	1.2	1.03 (0.41–2.59)	0.950
Recurrence or aggravation of symptomatic DVT	14 (3.0)	1.8	12 (2.5)	1.6	0.89 (0.41–1.92)	0.758
Acute coronary syndrome	0	-	3 (0.6)	0.4	-	0.078
Ischemic stroke	0	-	8 (1.7)	1.0	-	0.004
Death from any cause	40 (8.5)	4.9	45 (9.5)	5.8	1.16 (0.76–1.77)	0.500
Death related to VTE	4 (0.8)	0.5	2 (0.4)	0.3	0.52 (0.10–2.86)	0.448
Death related to CVD	6 (1.3)	0.7	5 (1.1)	0.6	0.86 (0.26–2.83)	0.810
Major bleeding	9 (1.9)	2.1	19 (4.0)	4.0	1.97 (0.89–4.36)	0.087
Minor bleeding	36 (7.6)	8.5	38 (8.0)	8.2	1.00 (0.64–1.58)	0.991
Clinically relevant events*	59 (12.5)	7.5	82 (17.2)	11.1	1.46 (1.05–2.04)	0.025

*Clinically relevant events were evaluated as a composite outcome, in which each component (recurrent VTE, acute coronary syndrome, ischemic stroke, death from any cause, and major bleeding events) was weighted equally. CI, confidence interval; CVD, cardiovascular disease; DVT, deep vein thrombosis; HR, hazard ratio; PE, pulmonary embolism; VTE, venous thromboembolism.

The rates of recurrence or aggravation of symptomatic VTE (0.4% per patient-year vs. not observed, respectively; *p* = 0.084) (Table 3; Figure 2C) and death from any cause (0.5% vs. 0.1% per patient-year, respectively; *p* = 0.181) tended to be higher within 30 days of starting therapy in both the high and low D-dimer groups, but the differences between the two groups were not significant. Additionally, the incidence of major bleeding within 30 days did not differ between the high and low D-dimer groups (0.9% vs. 0.4% per patient-year, respectively; *p* = 0.208). For the composite of clinically relevant events, there was a significant difference between the high and low D-dimer groups in the incidence of events occurring within 30 days (1.8% vs. 0.4% per patient-year, respectively; *p* = 0.013) (Table 3; Figure 2D).

**Table 3:** Landmark clinical outcomes according to baseline D-dimer concentration.

	Baseline D-dimer concentration				HR (95% CI)	*p* value
	Low *n* = 473		High *n* = 476			
	*n* (%)	Per patient-year	*n* (%)	Per patient-year		
**Recurrence or aggravation of symptomatic VTE**						
Within 30 days	0	0	3 (0.6)	0.40	-	0.084
Beyond 30 days	20 (4.3)	2.5	18 (3.9)	2.4	0.94 (0.49–1.77)	0.836
**Death from any cause**						
Within 30 days	1 (0.2)	0.1	4 (0.8)	0.5	3.98 (0.45–35.64)	0.181
Beyond 30 days	39 (8.3)	4.8	41 (8.8)	5.3	1.08 (0.70–1.68)	0.719
**Major bleeding**						
Within 30 days	3 (0.6)	0.4	7 (1.5)	0.9	2.32 (0.60–8.98)	0.208
Beyond 30 days	8 (1.7)	1.0	17 (3.7)	2.2	2.21 (0.96–5.13)	0.057
Clinically relevant events*						
Within 30 days	3 (0.6)	0.4	13 (2.7)	1.8	4.33 (1.23–15.19)	0.013
Beyond 30 days	56 (12.0)	7.1	69 (15.0)	9.4	1.30 (0.92–1.86)	0.138

*Clinically relevant events were evaluated as a composite outcome, in which each component (recurrent VTE, acute coronary syndrome, ischemic stroke, death from any cause, and major bleeding events) was weighted equally. CI, confidence interval; HR, hazard ratio; VTE, venous thromboembolism.

## 4. Discussion

This study has two major findings. First, patients with a high D-dimer concentration (≥7.6 μg/ml) were significantly older; more likely to have PE, proximal DVT, atrial fibrillation, or diabetes mellitus; and had a more severe condition, as indicated by a higher heart rate and a lower SpO_2_, than those with a low D-dimer concentration (<7.6 μg/ml). Patients with a high D-dimer concentration also more frequently underwent initial intensive therapy with rivaroxaban 30 mg/day. Second, the incidence of composite clinical events was significantly higher in the high D-dimer group than in the low D-dimer group, and in particular, as composite event items, the rates of major bleeding, ACS, and ischemic stroke were higher in the high D-dimer group.

### 4.1. Characteristics of patients with DVT and PE according to baseline D-dimer concentration

D-dimer concentration is often assessed to exclude the acute phase of DVT (17); therefore, DVT can be ruled out with high probability in patients with a D-dimer concentration below the reference value. Among the patients with VTE treated with rivaroxaban, PE, proximal DVT, atrial fibrillation, and diabetes mellitus were more common in the high D-dimer group than in the low D-dimer group. Similarly, a previous report showed that patients with proximal DVT and patients with PE with proximal DVT have significantly higher D-dimer concentrations than those with distal DVT (18).

A history of atrial fibrillation and diabetes mellitus in the high D-dimer group could be expected because cardiovascular risk factors, such as obesity, hypertension, dyslipidemia, diabetes mellitus, metabolic syndrome, and tobacco use, have been reported to increase the risk of VTE development (19).

### 4.2. Clinical outcomes of patients with DVT and patients with PE with/without DVT according to baseline D-dimer concentration

The high D-dimer group with VTE had a significantly higher rate of the composite endpoint than the low D-dimer group, with a particularly high rate of ischemic stroke. It is possible that these patients experienced ischemic stroke due to paradoxical embolism caused by patent foramen ovale, which is a common congenital abnormality observed in approximately 25% of the general population, with an even higher incidence of approximately 40% in the cryptogenic stroke population (20). It is widely known that some patients with non-valvular atrial fibrillation have elevated levels of molecular coagulation markers (21–23), especially clinically high-risk groups with CHADS_2_ scores exceeding 3 points. High D-dimer concentrations are associated with a significantly higher risk of systemic thromboembolism (24).

Although it has been shown that the relationship between non-valvular atrial fibrillation and PE is bidirectional, the detailed interrelationship between the two remains unclear (25). In this study, of the 8 patients who experienced ischemic stroke, none had a history of atrial fibrillation and 1 patient had diabetes mellitus. The 5 patients developed ischemic stroke despite receiving rivaroxaban, but the remaining 3 patients after the termination of rivaroxaban treatment. Therefore, the possibility that the patients had undiagnosed atrial fibrillation or that they developed atrial fibrillation during the observation period cannot be ruled out. In patients with high D-dimer concentrations, it is always necessary to determine the presence or absence of atrial fibrillation to rule out systemic thrombosis regardless of when rivaroxaban was administered.

Another considerable point is that the high D-dimer group in this study was older than the low D-dimer group, because aging has been reported to increase in D-dimer levels, the risk of developing VTE, and comorbidity complications (26). Thus, aging might have some effects on the incidence of the composite endpoint in this study.

The high D-dimer group with VTE had more severe cases of PE or proximal DVT, as can be seen by their baseline characteristics, and these patients were at a higher risk of systemic thromboembolism, which may have been the reason for the aggressive use of intensified therapy with rivaroxaban. In fact, many patients in the high D-dimer group with VTE underwent initial intensive therapy with rivaroxaban, the side effects of which may have increased the composite endpoint rate. In particular, careful attention should be paid to the high D-dimer group with VTE because they tended to have a 2-fold higher incidence of major bleeding than in the low D-dimer group. The rate of recurrent symptomatic VTE was not significantly different between the two groups, suggesting that rivaroxaban is effective in treating VTE regardless of the D-dimer concentration.

Within 30 days of treatment, recurrence or aggravation of symptomatic VTE and death from any cause tended to be higher in the high D-dimer group, but the difference was not significant. It is known that recurrence of symptomatic VTE is more common in the acute phase and that the incidence of death from any cause is increased as a result (27–30), and the present results are consistent with this.

D-dimer concentrations in patients with acute VTE are known to be related to the severity of the clot burden/thrombus extension, which may be related to early mortality and D-dimer concentration (31). In the COMMAND VTE Registry, a large-scale registry of patients with VTE in Japan, it was reported that patients within 30 days of VTE onset had a higher risk of VTE recurrence and death from any cause when the D-dimer concentration was high (32). Although the same trend was observed in the present study, the lack of significant differences between patients stratified by D-dimer concentration may be because of lower D-dimer concentrations, fewer patients with severe disease, more patients with DVT, and fewer patients with cancer than in the COMMAND VTE Registry. However, the present results suggest that D-dimer concentration may be an indicator of the composite endpoint in patients with VTE.

### 4.3. Clinical implications

Patients with higher baseline D-dimer concentrations may have non-valvular atrial fibrillation and/or diabetes mellitus, and have a high risk of systemic thromboembolism. Therefore, aggressive direct oral anticoagulant therapy in patients with high D-dimer concentrations who develop VTE can prevent recurrence or aggravation of symptomatic VTE. However, as the risk of major bleeding is also high during the period of intensified therapy, the choice of anticoagulation and the duration of administration should be adjusted according to the patient’s background.

This analysis alone is insufficient to elucidate a clear cut-off D-dimer concentration that separates bleeding events from thromboembolic events. Thus, further prospective studies are needed to clarify the prognostic value of D-dimer concentration in patients with VTE.

### 4.4. Limitations

This study has some limitations that should be noted. Although multiple studies have shown an association between elevated D-dimer concentration and prognosis, no single cut-off value has been identified that consistently optimizes the prognostic value of this biomarker, which this study did not overcome. Second, D-dimer concentration can change with age and comorbidities, such as atrial fibrillation, heart failure, peripheral arterial disease, and renal failure. Patients with these comorbidities were included in the present study, and the presence of such comorbidities may limit the diagnostic and prognostic value of D-dimer concentration specifically for VTE. For example, the D-dimer itself may not become a good prognostic predictor of future thrombotic and bleeding events if the population consists of a low incidence of atrial fibrillation and receiving a lower dose of rivaroxaban. Finally, only Japanese patients were included in this analysis, which may limit the generalizability of the findings.

## 5. Conclusion

In real-world clinical practice, Japanese patients with VTE with a high D-dimer concentration were significantly older and had higher rates of PE, proximal DVT, atrial fibrillation, and diabetes mellitus. Moreover, significantly more patients underwent initial intensive therapy with rivaroxaban 30 mg/day. Patients with VTE with a high baseline D-dimer concentration did not have significant differences in the incidence of recurrent or worsening symptomatic VTE, but they had a significantly higher rate of clinically relevant events (recurrent or worsening symptomatic VTE, ACS, ischemic stroke, death from any cause, and major bleeding) than the low D-dimer group, with a significant differences in the rate of cerebral infarction. Patients with VTE with a high D-dimer concentration may be a clinically high-risk group for systemic thromboembolism. These findings will help to manage anticoagulation therapy in terms of deciding the therapeutic duration and selecting between an intensive or preventative rivaroxaban dose.

## Data availability statement

The datasets presented in this article are not readily available because the deidentified participant data will not be shared. Requests to access the datasets should be directed to YO, okumura.yasuo@nihon-u.ac.jp.

## Ethics statement

The studies involving human participants were reviewed and approved by the Institutional Review Board of Nihon University Itabashi Hospital. All participating institutions also provided ethics approval. The patients/participants provided their written informed consent to participate in this study.

## Author contributions

SM: resources, investigation, visualization, writing–original draft, writing–review and editing. YO: conceptualization, resources, supervision, funding acquisition, investigation, visualization, project administration, writing–review and editing. IF: conceptualization, writing–review and editing. MN: conceptualization, writing–review and editing. NY: conceptualization, writing–review and editing. MT: conceptualization, resources, investigation, writing–review and editing. HM: conceptualization, resources, investigation, writing–review and editing. TaY: conceptualization, writing–review and editing. TI: conceptualization, resources, investigation, writing–review and editing. MM: conceptualization, writing–review and editing. TsY: conceptualization, writing–review and editing. AH: conceptualization, resources, investigation, supervision, funding acquisition, investigation, project administration, writing–review and editing. All authors contributed to the article and approved the submitted version.

## Funding

The authors declare that this study received funding from Bayer Yakuhin, Ltd. The funder was not involved in the study design, collection, analysis, interpretation of data, the writing of this article, or the decision to submit it for publication.

## Conflict of interest

YO received lecture fees from Bayer Yakuhin, Ltd., Bristol-Myers Squibb, AstraZeneca; lecture fees, scholarship funds, and donations from Daiichi-Sankyo Co., Ltd.; scholarship funds and donations from Nihon Medi-Physics; and is associated with endowed departments sponsored by Boston Scientific Japan, Abbott Medical Japan, Medtronic Japan Co., Ltd., Nihon Kohden Co., and Japan Lifeline Co., Ltd. NY has received lecture fees from Bayer Yakuhin, Ltd., Pfizer Japan Inc., and Daiichi-Sankyo Co., Ltd. TaY received lecture fees, manuscript fees, and research funding from Daiichi-Sankyo Co., Ltd., Bristol-Myers Squibb, and Bayer Yakuhin, Ltd., lecture fees from Ono Pharmaceutical Co., Ltd., Toa Eiyo, Ltd., Novartis Pharma KK, Otsuka Pharmaceutical Co., Ltd., and Nippon Boehringer Ingelheim Co., Ltd., and scholarship from Daiichi-Sankyo Co., Ltd. TI received lecture fees from Bayer Yakuhin, Ltd., Daiichi-Sankyo Co., Ltd., and Pfizer Japan Inc., and research funding from Daiichi-Sankyo Co., Ltd. MM received lecture fees from Bayer Yakuhin, Ltd. AH received lecture fees from Daiichi-Sankyo Co., Ltd. and Bayer Yakuhin, Ltd.

The remaining authors declare that the research was conducted in the absence of any commercial or financial relationships that could be construed as a potential conflict of interest.

## Publisher’s note

All claims expressed in this article are solely those of the authors and do not necessarily represent those of their affiliated organizations, or those of the publisher, the editors and the reviewers. Any product that may be evaluated in this article, or claim that may be made by its manufacturer, is not guaranteed or endorsed by the publisher.

## Supplementary material

The Supplementary material for this article can be found online at:

https://www.frontiersin.org/articles/10.3389/fcvm.2023.1074661/full#supplementary-material
