# Supplementary figures and images for: Relationship between baseline D-dimer and prognosis in Japanese patients with venous thromboembolism: Insights from the J’xactly study
Source: Front Cardiovasc Med. 2023 Feb 9;10:1074661. doi: 10.3389/fcvm.2023.1074661 (PMC9949378; doi:10.3389/fcvm.2023.1074661)

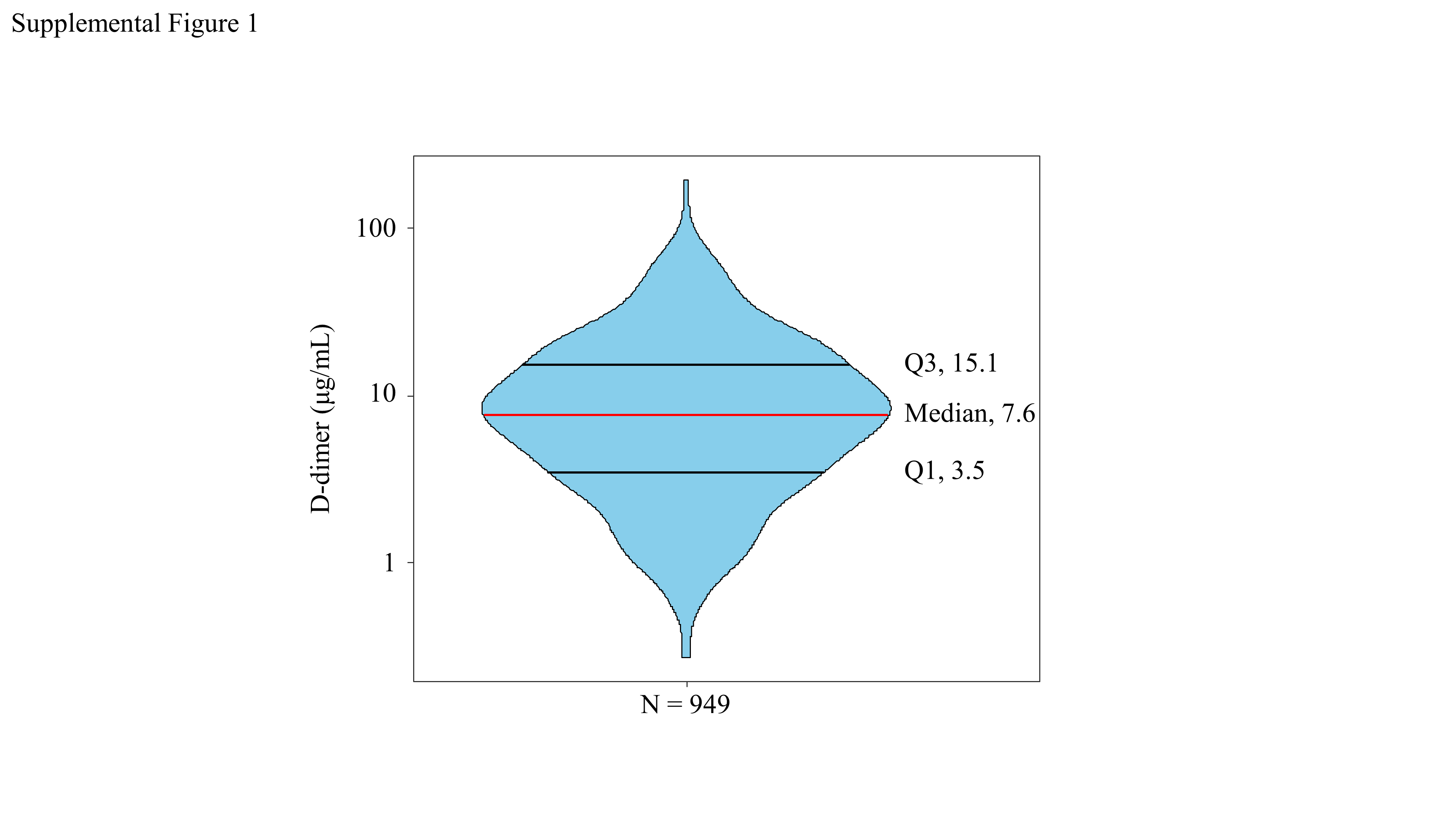

Supplement: Supplementary file 1 [file Image_1.tif]
